# Outcome of the incarcerated abdominal wall hernias managed by open and laparoscopic approaches

**DOI:** 10.12669/pjms.40.5.8899

**Published:** 2024

**Authors:** Hamed A. AlWadaani, Abdul Qadeer Memon

**Affiliations:** 1 Hamed A AlWadaani, MD, PhD. Department of Surgery, King Faisal University College of Medicine, Al-Ahsa 31982, Kingdom of Saudi Arabia; 2 Abdul Qadeer Memon, FCPS. Department of Surgery, King Faisal University College of Medicine, Al-Ahsa 31982, Kingdom of Saudi Arabia

**Keywords:** Hernia repair, Laparoscopy, Polypropylene mesh, TAPP, TEP

## Abstract

**Background & Objective:**

Laparoscopic surgery is generally considered as better than open surgery in terms of less complications, minimal hospital stays and quick healing of the wounds. Our objective was to compare the immediate and early outcome of the different incarcerated hernias of anterior abdominal wall operated on as emergency cases by open and laparoscopic approach.

**Methods:**

This is a retrospective comparative study which was conducted at two hospitals of AlAhsa city of the Eastern region of the Kingdom of Saudi Arabia from July, 2017 to June, 2022. The data were retrieved from the medical records of the hospitals. All male and female patients having different types of incarcerated hernias of anterior abdominal wall presenting to the emergency room were included in the study. The patients were divided in two groups; those who were operated on by open approach (Group-I) and those who were operated on by laparoscopic approach (Group-II).

**Results:**

Out of total 70 male and female patients, 42 were in Group-I and 28 in Group-II. The variety of the incarcerated hernias in both groups overall was para-umbilical 26(37.14%), incisional 18(25.71%), inguinal (right & left) 17(24.28%) and epigastric 9(12.86%). The mean operative time taken by Group I and II was 126.07 (±9.728) and 98.57 (±10.079) minutes respectively with a difference of 27.50 minutes (p=0.807). The mean hospital stay of the patients in Group I and II was 1.36(±0.719) and 1.57(±0.997) days respectively (p=0.482). The post-operative complications rate in Group-I was 6(14.28%) and in Group-II, 6(21.43%) (p=0.658). Overall, 12(17.14%) patients developed the complications in both groups. When the number of the complications is compared, it shows that there was no significant difference between the two groups (p=0.583).

**Conclusion:**

Laparoscopic approach is not superior to the open approach in the terms of the immediate and early outcome/complications of the incarcerated hernias of the anterior abdominal wall operated as emergency cases in this study.

## INTRODUCTION

“A hernia is the bulging of part of the contents of the abdominal cavity through a weakness in the abdominal wall”. The nomenclature of hernia varies according to the anatomical location. There are varieties of abdominal wall hernias, which include inguinal, femoral, umbilical, ventral, incisional hernias and many other rare types. A reducible hernia is that the contents of which are possible to reduce back into the abdomen. On the contrary, the contents of the sac of an irreducible or incarcerated hernia cannot be returned to the abdomen. The strangulation and obstruction are other possible life-threatening complications. That is why, such hernias (incarcerated) need emergency surgical management to save the viability of the contents of the sac along with the repair of the defects to prevent recurrence. Delay in the presentation of hernias may lead to the complications and significantly increases the morbidity and risk of mortality.^1^

Laparoscopic surgery is generally considered superior to the open surgery because this approach reduces the hospital stay and the number of other complications due to less trauma to the tissues and less blood loss in the expert hands as compared to open surgery.^2^ Different abdominal wall hernias are repaired by different procedures through open or laparoscopic approaches. Laparoscopic hernia repair may be sometimes challenging in patients having abdominal obesity.^3^ Umbilical hernia is repaired by Mayo technique or by putting mesh via open or laparoscopic approach. For inguinal hernias, Shouldice, Lichtenstein and many other open techniques or total extra-peritoneal (TEP) and transabdominal pre-peritoneal (TAPP) by laparoscopic approach are used. Recurrence of hernia is the main concern of the surgeons along with other postoperative complications e.g. seroma/hematoma formation, urinary retention, neuralgias, testicular pain and swelling, mesh and wound infection etc., which determines the choice of the surgical procedure and the approach. Hence, abdominoplasty in the hernia patients is performed to achieve the primary musculo-fascial closure reinforced with polypropylene mesh.^4^

## METHODS

This retrospective comparative study was conducted at two hospitals of AlAhsa city of the Eastern region of the Kingdom of Saudi Arabia from July 2017 to June 2022. The data of the patients were retrieved from the medical records of the hospitals, then recorded and analyzed using IBM-SPSS-22. Descriptive statistics, such as mean and standard deviation, were calculated for quantitative variables. Qualitative results were presented as frequency and percentage, with tables. Pearson Chi-square test was conducted to find out the significance of the early and immediate outcome. Comparison in general data was set statistically significant if P < 0.05. All male and female patients having different types of incarcerated hernias of anterior abdominal wall presenting to the emergency room (ER) during the above period were included in the study. The patients were divided in two groups; those who were operated on by open approach (Group-I) and those who were operated on by laparoscopic approach (Group-II). The patients who were converted from the laparoscopic approach to the open approach were considered as open (Group-I). The hernias of both groups were repaired by polypropylene (PP) mesh.

### Ethical Approval

The study was approved by the Institutional Ethics Committee of King Faisal University (Ref No. 2020-12-45 dated 27/12/2020).

### Surgical Procedure

In the laparoscopic approach, the procedure depends upon the type of hernia. For the inguinal hernias, transabdominal pre-peritoneal (TAPP) technique was used. A 10 mm camera port was inserted at infra-umbilical area. Two 5mm ports were inserted at midclavicular line on each side, where the distance was adjusted according to the site of hernia and size of the abdomen. After other usual steps, the mesh was inserted through the camera port. For other anterior abdominal wall hernias, the 10mm camera port was inserted at the infra-umbilical area. The three-port-technique was used entering at the other two sites at the anterior axillary line in the hypochondrium and the iliac regions. In the open approach, the Lichtenstein procedure was adopted for inguinal hernias to insert the PP mesh and on-lay mesh for the para-umbilical and incisional hernias.

## RESULTS

Out of total 70 patients, 53(75.7%) were females and 17(24.3%) were males. Their age range was between 18 and 72 years with mean age of 43.10 years (±12.808) (p=0.525). There were 42(60%) patients in Group-I and 28(40%) in Group-II. The overall male female ratio in both groups was 1:3 (p=0.866). Thirty-nine patients were actually initiated with laparoscopic approach; out of which, 11(28.2%) patients were converted from laparoscopic to open approach due to severe adhesions, bowel ischemia and intestinal perforation. These 11 patients were hence placed in group-I and the remaining 28 patients were placed in group-II.

The variety of the incarcerated hernias in both groups overall was para-umbilical 26(37.14%), incisional 18(25.71%), inguinal (right & left) 17(24.28%) and epigastric 9(12.86%) (Table-I). One (1.43%) female patient with epigastric hernia had uncontrolled diabetes mellitus. As both groups are compared, the mean operative time taken by Group-I was 126.07 (±9.728) minutes, while that of Group-II was 98.57 (±10.079) minutes (p=0.807). The mean hospital stay of the patients in Group-I was 1.36(±0.719) days and in Group-II was 1.57(±0.997) days (p=0.482). One (2.4%) patient in Group-I was re-admitted due to uncontrolled diabetes mellitus (DM) and none was re-admitted in Group-II (p=0.416).

**Table-I:** Biodata of the patients.

Type of hernia	Group-I (N=42) (Open approach) n (%) Male: Female	Group-II (N=28) (Laparoscopic approach) n (%) Male: Female	Mean Age (Years) (Std. Dev)
Gender	10(23.81):32(76.19)	07(25):21(75)	--
Age Overall (Years)	Mean=44.50 (±13.095)	Mean=41.00 (±12.098)	--
	Median=42.50	Median=41.50	
	Range= 21-72	Range= 18-65	
Para-umbilical (n=26) (37.14%)	00(00):15(35.71)	00(00):11(39.28)	39.38(±10.273)
Incisional (n=18) (25.71%)	01(2.38):11(26.19)	01(3.57):05(17.86)	45.39(±14.353)
Epigastric (n=9) (12.86%)	01(2.38):04(9.52)	00(00):04(14.28)	43.44(±9.153)
Right inguinal (n=12) (17.14%)	05(11.90):02(4.76)	05(17.86):00(00)	44.50(±13.070)
Left inguinal (n=5) (7.14%)	03(7.14):00(00)	01(3.57):01(3.57)	50.20(±21.788)
Total=70(100%)	42(60)	28(40)	43.10 (±12.808)

**Fig.1:**
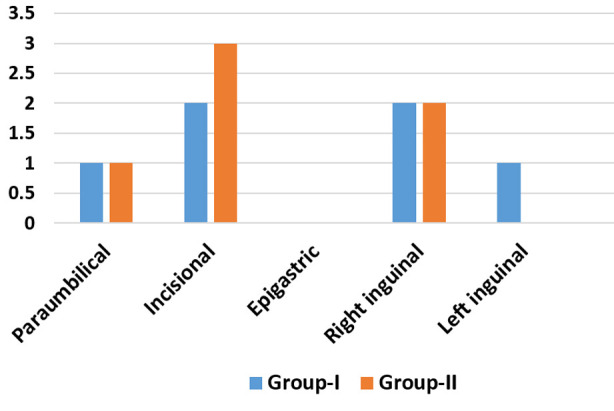
Comparison of complications according to the type of hernia.

The post-operative complications rate in Group-I was 6(14.28%) and in Group-II, 6(21.43%). Two patients in each group had bowel resection along with the hernia repair (Table-II) (p=0.658). Different post-operative complications were observed in both the groups which included the wound infection, scrotal hematoma, seroma, urinary retention, upper respiratory tract infection (URTI), aspiration pneumonia and gastro-intestinal (GI) perforation. (Table-III). When the number of the complications is compared, it shows that both the groups have the same number (Fig.1) (p=0.583).

**Table-II:** Results.

Outcome	Group-I (Open approach) (n=42)	Group-II (Laparoscopic approach) (n=28)
Operative time (Minutes)	Mean=126.07 (±9.728)	Mean=98.57 (±10.079)
	Median=125.00	Median=95.00
Hospital Stay (Days)	Mean=1.36(±0.719)	Mean=1.57(±0.997)
	Median=1 day	Median=1 day
	1 day=32(76.3%)	1 day=18(64.3%)
	2 days=6(14.3%)	2 days=7(25.0%)
	3 days=3(7.1%)	3 days=1(3.6%)
	4 days=1(2.4%)	4 days=1(3.6%)
	--	5 days=1(3.6%)
Readmission	01(2.4%)	00
Conversion	Not applicable	39-28=11(28.20%)
Complications	06(14.28%)	6(21.43%)
Additional Procedure	Bowel resection = 2(4.76%)	Bowel resection = 2(7.14%)
Total = 70	42	28

**Table-III:** Types of complications and their frequencies.

Complication (n=70)	Group-I (Open approach) (n=42)	Group-II (Laparoscopic approach) (n=28)
Wound infection	1(2.4%)	2(7.1%)
Scrotal hematoma	1(2.4%)	2(7.1%)
Seroma	1(2.4%)	00
Urine retention	1(2.4%)	00
URTI	2(4.7%)	00
Aspiration pneumonia	00	1(3.6%)
GI Perforation	00	1(3.6%)
	6(14.28%)	6(21.43%)

## DISCUSSION

Minimal invasive (laparoscopic) surgery has become order of the day now a days. PP mesh with different varieties are frequently used to repair hernias.^5^ It is suggested that the laparoscopic approach reduces the hospital stay and the number of complications due to less trauma to the tissues and less blood loss in the expert hands as compared to open surgery. However, there is also a different opinion about the outcome of the laparoscopic surgery in inguinal hernia.^2^ Most of the anterior abdominal wall hernias present as outpatient cases without complications and are operated electively. Emergency cases present with complications, though less in number.

Most of the patients in this study are females (M:F = 1:3). The data show that most of the para- umbilical and incisional hernia patients are females and the inguinal hernia patients are males. However, this ratio is different in different countries, but the influencing factors may be obesity, previous abdominal surgery or trauma, family history and grand multipara etc. In Saudi Arabia, obesity and multipara women may be the predominating factors.^6^,^7^ Gender has no significant effect on the outcome in this study (p=0.866).

Mean and median age of the patients in both the groups was within forties. It shows that the complicated hernias are mostly present in their fifth and sixth decades of life (Table-I). Mean/Median age of the patients in both groups was 44.50/42.50 and 41.00/41.50 respectively. Same is the pattern of age observed in different studies conducted by Pandya B et al. and Olasehinde O et al.^6^,^8^ The age does not show the difference in outcome in this study (p=0.525).

There is no significant difference in the operative time in both of the approaches in the expert hands. However, the conversion if needed, increases the operative time to a little extent. In this study, the mean operative time in Group-I and Group-II was 126.07 (±9.728) and 98.57 (±10.079) minutes respectively with a difference of 27.5 minutes. Different studies present different statistical data about the operation time. Alchalabi H et al. in his review study shows borderline difference of 15 minutes in both of the groups, while Rogmark shows laparoscopic operation quicker than open and Pring shows no difference.^9^ Eleven (28.2%) out of 39 patients who were initially selected for laparoscopic approach in this study needed conversion from laparoscopic to the open approach due to difficult adhesions, questionable viability of the bowel. Hence, if converted, the laparoscopic approach takes more time than open but overall, it takes lesser than the open approach (p=0.807) (Table-II). However, the converted cases are considered as open approach in this study.

Two patients in each group had bowel resection as an additional surgical procedure along with the mesh repair. The overall conversion rate in the uncomplicated hernias with elective hernia repairs is 4.05% and independent risk factors may include large hernia defect, previous abdominal surgery, previous hernia surgery and scrotal hernia.^10^ Learning curve is another factor of the conversion. In another study of inguinal hernias repair electively by TEP, the conversion rate is 11%.^11^ Therefore, the higher conversion rate in this study is justifiable because of complicated hernias operated as emergency cases. The choice of the surgical approach (open or laparoscopic) has a little significance in this study (p=0.416). One (2.3%) patient needed readmission due to uncontrolled DM postoperatively. The patient was switched to the short acting insulin and the DM became under control.

Both the groups had common immediate and early postoperative complications like wound infection, scrotal hematoma, seroma, urinary retention, upper respiratory tract infection and aspiration pneumonia. However, one (3.6%) patient in the Group-II developed GI perforation during operation and was converted to the open approach. In this study, the rate of complications in Group-I is 6(14.28%) and in Group-II, it is 6(21.43%). The number of such patients in both of the groups is the same. The overall complications rate is 12(17.14%) (Table-III). The rate of the complications in this study is quite acceptable as compared to other studies. Dai W et al. in their study of emergency repair of incarcerated groin hernia has overall postoperative complications rate equal to 40.6%.^12^ All these complications are reported in the literature. Late complications like mesh infections, adhesions, recurrence etc. may be expected when the patients are followed-up for longer period.^13^,^14^ There is no significant difference in the complications of both the approaches (p=0.583) (Fig.1). Meier J et al. and Moreno-Suero F et al. have observed the same outcome in their studies.^2^,^15^ The independent risk factors of hernia complications may include large defect of hernia, complicated hernias presented to emergency, obesity and other concomitant systemic diseases like diabetes mellitus, respiratory or urinary tract infections etc.

### Limitations of the study

Most of the anterior abdominal wall hernias present as outpatient cases without complications and are operated electively, while the number of complicated hernias presenting to emergency is less. Hence, the number of the patients in the study is less even extending to many years. These are the limitations of this study that should be acknowledged. The sample size was relatively small, which limits the statistical power to detect smaller differences between the groups. Furthermore, the follow-up period of the patients available in the record for this study was less, hence immediate and early complications are noted. Longer-term follow-up could provide more information about the late complications and outcomes.

## CONCLUSION

This study shows that the laparoscopic approach is not superior to the open approach in the terms of the immediate and early outcome/complications of the incarcerated hernias of the anterior abdominal wall operated as emergency cases.

### Recommendation

Any approach (open or laparoscopic) is suitable with the same results to treat the incarcerated hernias of the anterior abdominal wall depending upon the facilities available at the set-up.

### Declaration

The first/corresponding author is a senior and expert surgeon having an extensive experience in open as well as minimal invasive surgery.

### Authors’ Contribution:

**AH:** Searched, collected, analyzed the data, edited, reviewed and finally approved the manuscript.

**MA:** Searched and collected the data, analyzed and wrote the manuscript.
